# Adverse reaction profiles of hemorrhagic adverse reactions caused by direct oral anticoagulants analyzed using the Food and Drug Administration Adverse Event Reporting System (FAERS) database and the Japanese Adverse Drug Event Report (JADER) database

**DOI:** 10.7150/ijms.34629

**Published:** 2019-09-07

**Authors:** Kazuyo Shimada, Shiori Hasegawa, Satoshi Nakao, Ririka Mukai, Sayaka Sasaoka, Natsumi Ueda, Yamato Kato, Junko Abe, Takayuki Mori, Tomoaki Yoshimura, Yasutomi Kinosada, Mitsuhiro Nakamura

**Affiliations:** 1 Laboratory of Drug Informatics, Gifu Pharmaceutical University; 1-25-4 Daigaku-Nishi, Gifu 501-1196, Japan; 2 Medical Database Co., Ltd., 3-11-10 Higashi, Shibuya-ku, Tokyo, 150-0011, Japan; 3 Department of Pharmacy, Ogaki Municipal Hospital, 4-86 Minaminokawa-cho, Ogaki, Gifu, 503-8502; 4 United Graduate School of Drug Discovery and Medical Information Sciences, Gifu University, 1-1 Yanagido, Gifu, 501-1194, Japan; Current Address

**Keywords:** direct oral anticoagulant, hemorrhage, adverse reaction, FAERS, JADER

## Abstract

Direct oral anticoagulants (DOACs) are used in anticoagulant therapy. The purpose of this study was to evaluate the association of DOAC-induced gastrointestinal (GI) and nervous system hemorrhage using the FDA's Adverse Event Reporting System (FAERS) database and the Japanese Adverse Drug Event Report (JADER) database.

We identified and analyzed the reports of hemorrhagic reactions between 2004 and 2016 from the FAERS and JADER databases, and calculated the adjusted reported odds ratio (ROR) using the multiple logistic regression method. Additionally, we used the time-to-onset analysis.

In the FAERS database, the adjusted ROR of apixaban, rivaroxaban, and dabigatran for GI hemorrhage was 6.79 (5.84-7.91), 19.58 (18.85-20.34), and 14.51 (13.58-15.51), respectively. In the JADER database, the adjusted ROR of apixaban, rivaroxaban, edoxaban, and dabigatran for GI hemorrhage was 11.80 (9.50-14.64), 11.03 (9.18-13.26), 10.17 (6.95-14.88), and 9.85 (7.23-13.42), respectively. We found that the association of GI hemorrhage with DOACs was affected by sex (female). Additionally, 30% of GI hemorrhage was observed after 30 days.

Hemorrhagic reactions of both GI and nervous systems were observed in both the spontaneous reporting system databases. We recommend that female patients who experience symptoms related to GI hemorrhage should be closely monitored and advised to adhere to an appropriate care plan. Additionally, our results show that patients should be closely monitored for hemorrhage even after a month.

## Introduction

Direct oral anticoagulants (DOACs) are used for anticoagulant therapy to prevent stroke associated with atrial fibrillation and for the prevention and treatment of venous thromboembolic disease 1-4. DOACs directly inhibit thrombin (dabigatran 5) or factor Xa (rivaroxaban 6, apixaban 7, and edoxaban 8) to exert their anticoagulant effect. DOACs have advantages over vitamin K antagonists (e.g., warfarin), such as a more rapid anticoagulant effect, fewer individual differences in therapeutic effect, fixed-dose administration, less drug-drug interactions, and limited dietary restrictions 1-4,9. Randomized clinical trials (dabigatran (Randomized Evaluation of Long-Term Anticoagulation Therapy (RE-LY) trial) 1, rivaroxaban (Rivaroxaban Once-daily oral direct factor Xa inhibition Compared with vitamin K antagonism for prevention of stroke and Embolism Trial in Atrial Fibrillation (ROCKET AF) trial) 2, apixaban (Apixaban for Reduction in Stroke and Other Thromboembolic Events in Atrial Fibrillation (ARISTOTLE) trial) 3, and edoxaban (Effective Anticoagulation with Factor Xa Next Generation in Atrial Fibrillation-Thrombolysis in Myocardial Infarction study 48 (ENGAGE AF TIMI-48) trial) 4) have demonstrated that DOACs are associated with lower risk of intracranial hemorrhage than that with warfarin.

Renal function is an important factor in DOAC therapy, because each DOAC has varying degrees of renal elimination. The urinary excretion rate of dabigatran, rivaroxaban, apixaban, and edoxaban is reported to be 80% 10, 36% 11, 27% 12, and 50% 13,14, respectively. Moderate to severe renal impairment could increase the risk for hemorrhage due to the accumulation of drugs in the serum, affecting dabigatran the most and apixaban the least 15,16. Renal impairment is more often observed among elderly patients compared with that in the general population. Because atrial fibrillation is largely a disease of the elderly population, the risk of stroke and hemorrhage with DOAC therapy increases with age. The possible association of DOAC with gastrointestinal (GI) hemorrhage is of interest in elderly patients. Because ischemic strokes and systemic embolisms have greater clinical significance than nonfatal hemorrhage (e.g., GI hemorrhage), higher doses of DOACs (e.g., dabigatran) are more favorable in elderly patients 1. However, both acute and chronic GI hemorrhages have a negative effect on the patient's quality of life. A limitation of the standard-dose DOACs is an increase in the risk of GI hemorrhage 17-20. Moreover, there have been concerns regarding the safety profile of DOACs relating to age and sex; for example, renal functions are affected by sex 21, 22.

To monitor the adverse drug reactions, a spontaneous reporting system (SRS) compiles reports of suspected adverse reactions (ARs) either voluntarily reported by patients, clinicians, pharmacists, and other healthcare professionals or mandatorily reported by various pharmaceutical manufacturers. SRS is a valuable tool in post-marketing surveillance that reflects the realities of clinical practice 23. The US Food and Drug Administration (FDA) has developed the FDA Adverse Event Reporting System (FAERS). The Pharmaceuticals and Medical Devices Agency (PMDA), a regulatory authority in Japan, has developed the Japanese Adverse Drug Event Report (JADER) database.

Several researchers have evaluated hemorrhage risk associated with dabigatran using the FAERS database 24-26. Southworth et al. 24 demonstrated that dabigatran and warfarin were associated with similar hemorrhage rates, which is consistent with the findings of the RE-LY study. McConeghy et al. 25 reported that the reporting rate of hemorrhage related to dabigatran and warfarin was 26% and 32%, respectively. They also reported that intracranial hemorrhage was low with dabigatran, but the rate of GI hemorrhage was increased compared to that with warfarin, both of which are consistent with the findings of RE-LY. We previously demonstrated that GI hemorrhage was significantly increased in patients over the age of 80 years 26. Despite the insights that these trials provide, the effects of other DOACs on hemorrhagic ARs in a clinical setting are uncertain.

In this study, we evaluated the relationship between DOACs and hemorrhagic ARs using the reporting odds ratio (ROR) adjusted using the multiple logistic regression analysis 26-29. Analysis of time-to-onset data has been proposed as a method to detect signals for ARs in SRS. We also analyzed the time-to-onset of hemorrhagic ARs 30-32.

## Methods

### Data source

All data from the SRS database were fully anonymized by the regulatory authorities before we used them. Data from January 2004 to December 2016 in the FAERS database are publicly available and can be downloaded from the FDA website (http://www.fda.gov/). The FAERS database permits contributors to register drugs under any name, including a trade name and an abbreviation. The DrugBank database contains information of drugs used globally, including the FDA-approved small molecule drugs; it was utilized as a resource for batch conversion and compilation of drug names 33. We followed the FDA's recommendation to adopt the most recent CASE number to identify duplicate reports of the same patient from different reporting sources and excluded them from the analysis 34.

Data from April 2004 to November 2016 in the JADER database were extracted from the PMDA website (www.pmda.go.jp). We built a database that integrated data from the FAERS and JADER databases and the DrugBank using FileMaker Pro 13 software (FileMaker, Santa Clara, CA, U.S.A.) following the international safety reporting guidelines (International Council on Harmonization, E2B).

### Definition of hemorrhage reactions

The AR definitions used in this study corresponded to those provided by the Medical Dictionary for Regulatory Activities (MedDRA)/Japanese version 19.0 (MedDRA/J, www.pmrj.jp/jmo/php/indexj.php) 35. To evaluate the effect of DOACs on hemorrhagic reactions, we used a standardized MedDRA inquiry (SMQ) for hemorrhage reactions (SMQ code: 20000039) and the System Organ Class (SOC) for GI disorder, and extracted only reports that met both criteria. The number of selected preferred terms for hemorrhagic reactions, limited by SOC (GI disorder), was 152. Furthermore, to evaluate the nervous system hemorrhage, we used 88 preferred terms that matched the SMQ for hemorrhage reactions (SMQ code: 20000039) and the SOC for the nervous system disorder.

### Analysis

To evaluate the effect of age on “hemorrhagic reactions,” the reports were stratified into the following age groups: 0-59 years and more than 60 years. According to the definition of the World Health Organization (WHO) of the United Nations, elderly people are those who are aged 65 years or more.

Using established pharmacovigilance indices 23, we evaluated the ROR to establish the effects of DOACs on “hemorrhagic reactions.” “Cases” were defined as patients who reported “hemorrhagic reactions,” while “non-cases” consisted of patients associated with all other reports. The ROR is the ratio of the odds of reporting ARs versus all other reactions associated with DOACs compared with the reporting odds for all other drugs present in the database. To compare the “cases” and “non-cases,” we calculated the RORs as (a:c) / (b:d). The RORs were expressed as point estimates with a 95% confidence interval (CI). The signal was considered positive if the lower limit of 95% CI was > 1 and the reported number was ≥ 2 36.

The use of ROR allows adjustment using multiple logistic regression analysis and provides the advantage of controlling covariates 37,38. In this analysis, the results were refined by dedicated correction to detect confounding factors that may be present in the database. We calculated the adjusted ROR to control the covariates using the multiple logistic regression analysis. The report was stratified according to age as follows: 0-59- and ≥ 60-year-old group. To construct a multiple logistic model that coded report year, sex, stratified age group, and drug, the following multiple logistic model was used for analysis:


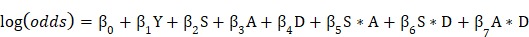


(Y = reporting year, S = sex, A = stratified age group, and D = drug (apixaban, rivaroxaban, edoxaban, and dabigatran)).

The adjusted ROR was calculated using the 0-59-year-old group as the control group. The effectiveness of explanatory variables was evaluated using a stepwise method with a significance level of 0.05 (forward, and backward) 27,28. Using the likelihood ratio test, the influence of explanatory variables was evaluated. As the difference of -2log likelihood follows chi-square distribution with one degree of freedom, the results with p ≤ 0.05 were considered statistically significant. Data analysis was performed using JMP software version 12.0 (SAS Institute Inc., Cary, NC, USA).

Time-to-onset duration was calculated from the time of a patient's first prescription to the occurrence of hemorrhagic reactions. The records with completed AR occurrence and prescription start date were used for the time-to-onset analysis. It was necessary to consider right truncation when evaluating the time-to-onset of ARs. We determined an analysis period of 365 days after the start of administration. The median duration, quartiles, and Weibull shape parameters (WSPs) were used to evaluate the time-to-onset data. The scale parameter α of Weibull distribution determines the scale of the distribution function. A larger scale value (α) stretches the distribution, whereas a smaller scale value (α) shrinks data distribution. The WSP β of Weibull distribution determines the shape of distribution function. Larger and smaller shape values produce left- and right-skewed curves, respectively. The shape parameter β of Weibull distribution was used to indicate the level of hazard over time without a reference population. When β is equal to 1, the hazard is estimated to be constant over time. If β is greater than 1 and 95% CI of β excluded the value 1, the hazard was considered to increase with time 30,31,39. The time-to-onset analysis was performed using JMP software version 12.0 (SAS Institute Inc., Cary, NC, USA).

## Results

The FAERS database contained 8,867,135 AR reports from January 2004 to December 2016. After excluding duplicates according to the FDA's recommendation, 7,348,357 reports were analyzed. The JADER database contained 430,587 reports from April 2004 to November 2016.

### Gastrointestinal hemorrhage reaction

The reporting rate of GI hemorrhage related to apixaban, rivaroxaban, edoxaban, and dabigatran was 9.5%, 24.5%, 23.8%, and 22.2% in FAERS and 23.1%, 21.5%, 27.8%, and 25.7% in JADER, respectively (Table 1).

For the FAERS database, the crude ROR with 95% CI for GI hemorrhage for apixaban, rivaroxaban, edoxaban, and dabigatran was 5.83 (5.54-6.13), 20.04 (19.65-20.43), 17.18 (10.27-28.75), and 16.90 (16.51-17.30), respectively (Table 1). The crude ROR (95% CI) for dabigatran in 0-59-year-old group and ≥ 60-year-old group was 10.41 (9.29-11.66) and 19.52 (18.95-20.11), respectively (Table 1).

For the JADER database, the crude ROR with 95% CI for apixaban, rivaroxaban, edoxaban, and dabigatran was 12.24 (11.12-13.46), 11.20 (10.22-12.27), 15.14 (12.48-18.35), and 13.96 (12.55-15.52), respectively (Table 1). The crude ROR (95% CI) for dabigatran in 0-59-year-old group and ≥ 60-year-old group was 4.19 (2.02-8.70) and 14.66 (13.15-16.34), respectively (Table 1).

After excluding incomplete reports that lacked information on the report year, age and sex, 4,383,074 reports in FAERS and 398,645 reports in JADER were included in the multiple logistic regression analysis. Using a stepwise logistic regression model, we selected significant variables related to ARs among the reporting year, age, sex, and administered drugs (apixaban, rivaroxaban, edoxaban, and dabigatran), and examined the interaction between sex, age, and the administered drug (Table 2, Table S1).

For the FAERS database, the result of the final model indicated that reporting year (p < 0.0001), age (≥ 60 years, p < 0.0001), sex (female, p < 0.0001), and the administered drug [apixaban (p < 0.0001), rivaroxaban (p < 0.0001), and dabigatran (p < 0.0001)] had significant effects (Table 2). The adjusted ROR of apixaban, rivaroxaban, and dabigatran was 6.79 (5.84-7.91), 19.58 (18.85-20.34), and 14.51 (13.58-15.51), respectively. The interaction of age (≥ 60)*sex (female) (p < 0.0001), sex (female)*apixaban (p < 0.0001), sex (female)*rivaroxaban (p < 0.0001), and sex (female)*dabigatran (p < 0.0001) was also significant. The adjusted ROR for sex (female)*apixaban, sex (female)*rivaroxaban, and sex (female)*dabigatran was 1.40 (1.24-1.59), 1.36 (1.29-1.43), and 1.52 (1.44-1.62), respectively.

For the JADER database, significant contributions were observed for reporting year (p < 0.0001), age (≥ 60 years, p < 0.0001), sex (female, p < 0.0001), and the administered drug [apixaban (p < 0.0001), rivaroxaban (p < 0.0001), edoxaban (p < 0.0001), and dabigatran (p < 0.0001)] (Table 2). The adjusted ROR of apixaban, rivaroxaban, edoxaban, and dabigatran was 11.80 (9.50-14.64), 11.03 (9.18-13.26), 10.17 (6.95-14.88), and 9.85 (7.23-13.42), respectively. The adjusted ROR for age (≥ 60)*dabigatran (p = 0.0380), sex (female)*apixaban (p < 0.0001), sex (female)*rivaroxaban (p < 0.0001), and sex (female)*dabigatran (p < 0.0001) was 2.29 (1.05-5.01), 1.60 (1.31-1.97), 1.79 (1.47-2.17), and 2.01 (1.61-2.51), respectively.

**Figure 2:**
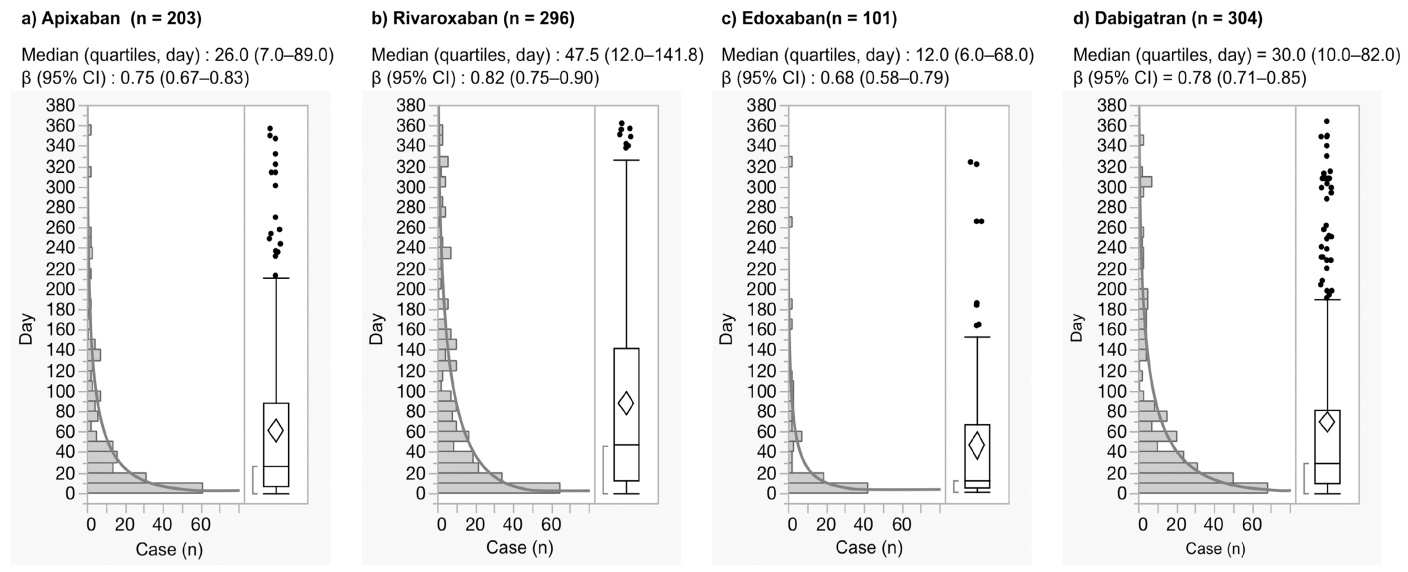
Histogram and Weibull shape parameter of gastrointestinal hemorrhage.

The time-to-onset profiles in the JADER database are demonstrated in Fig. 2. The median and quartiles of GI hemorrhage after the use of apixaban, rivaroxaban, edoxaban, and dabigatran were 26.0 (7.0-89.0), 47.5(12.0-141.8), 12.0 (6.0-68. 0), and 30.0 (10.0-82.0) days, respectively (Fig. 2). GI hemorrhage during the first 30 days after the use of apixaban, rivaroxaban, edoxaban, and dabigatran was 52.2%, 40.9%, 62.4%, and 49.0%, respectively.

### Nervous system hemorrhage reaction

The reporting rate of nervous system hemorrhage related to apixaban, rivaroxaban, edoxaban, dabigatran was 4.7%, 6.0%, 3.8%, 5.0% in FAERS and 24.8%, 23.2%, 17.1%, 12.4% in JADER, respectively (Table 1).

For the FAERS database, the crude ROR with 95% CI for nervous system hemorrhage for apixaban, rivaroxaban, edoxaban, and dabigatran was 11.23 (10.47-12.04), 15.63 (15.08-16.20), 8.65 (2.73-27.41), and 12.42 (11.88-12.98), respectively (Table 2). The crude ROR (95% CI) for dabigatran in 0-59-year-old group and ≥ 60-year-old group was 8.91 (7.18-11.05) and 13.73 (12.98-14.52), respectively (Table 1).

For the JADER database, the crude ROR with 95% CI for apixaban, rivaroxaban, edoxaban, and dabigatran was 23.59 (21.46-25.93), 21.71 (19.83-23.76), 13.55 (10.77-17.05), and 9.50 (8.25-10.93), respectively (Table 1).

Using a stepwise logistic regression model, important variables relevant to GI hemorrhage and nervous system hemorrhage were selected (Table 2). For FAERS, reporting year (p < 0.0001), age (≥ 60 years, p < 0.0001), sex (female, p < 0.0001), and the administered drug [apixaban (p < 0.0001), rivaroxaban (p < 0.0001), and dabigatran (p < 0.0001)] showed significant effect. The adjusted ROR for apixaban, rivaroxaban, and dabigatran was 13.64 (10.94-17.01), 15.46 (14.31-16.70), and 11.75 (10.34-13.34), respectively. The interaction of age (≥ 60)*sex (female) (p < 0.0001), age (≥ 60)*rivaroxaban (p = 0.0455), age (≥ 60)*dabigatran (p = 0.0162), sex (female)*apixaban (p < 0.0001), sex (female)*rivaroxaban (p < 0.0001), sex (female)*dabigatran (p < 0.0001) was significant. The adjusted ROR for age (≥ 60)*sex (female), age (≥ 60)*rivaroxaban, age (≥ 60)*dabigatran, sex (female)*apixaban, sex (female)*rivaroxaban, and sex (female)*dabigatran was 1.15 (1.09-1.22), 1.15 (1.00-1.32), 0.76 (0.61-0.95), 1.65 (1.39-1.96), 1.29 (1.18-1.41), and 1.37 (1.22-1.53), respectively.

For JADER, the adjusted ROR of apixaban (p < 0.0001), rivaroxaban (p < 0.0001), edoxaban (p < 0.0001), and dabigatran (p < 0.0001) was 34.07 (28.02-41.42), 29.58 (25.07-34.90), 17.63 (12.47-24.94), and 8.24 (5.48-12.40), respectively (Table 2).

**Figure 3:**
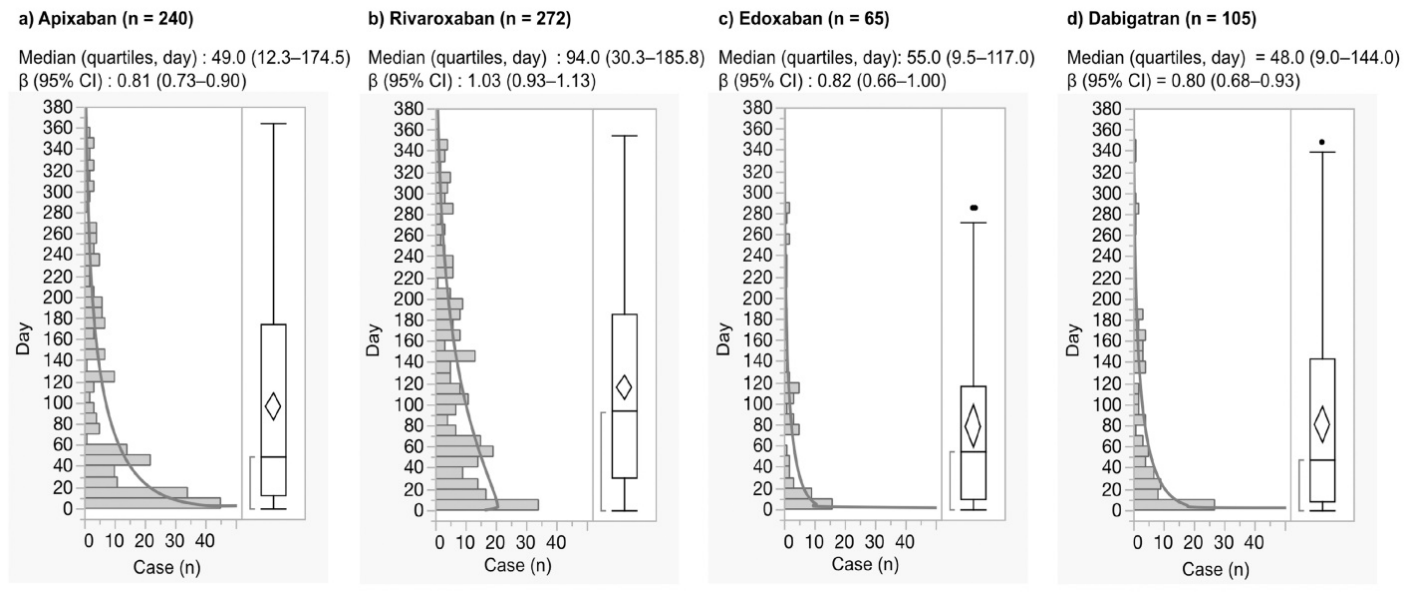
Histogram and Weibull shape parameter of nervous system hemorrhage.

For the JADER database, the median and quartiles of nervous system hemorrhage after the use of apixaban, rivaroxaban, edoxaban, and dabigatran were 49.0 (12.3-174.5), 94.0 (30.3-185.8), 55.0 (9.5-117.0), and 48.0 (9.0-144.0) days, respectively (Fig. 3). Nervous system hemorrhage within the first 30 days after the use of apixaban, rivaroxaban, edoxaban, and dabigatran was 37.5%, 23.9%, 43.1%, and 41.9%, respectively.

## Discussion

In this study, we evaluated the association between DOAC and hemorrhagic reactions using data from the SRS databases. As the crude RORs of hemorrhagic reactions such as GI hemorrhage and nervous system hemorrhage were higher than one in both the SRS databases, our results suggest that DOACs increase the adverse hemorrhagic reactions.

Renal function is affected by age, sex, body weight, clinical condition, and medication. The effect of age on ARs associated with DOAC therapy has been widely reported. We applied the multiple logistic regression analysis to validate the results. The interaction term of age (≥ 60)*dabigatran for GI hemorrhage was significant in the JADER databases. Dabigatran presented high renal excretion rate 21. Because the renal excretion may be compromised, GI hemorrhage was increased in the elderly patients 21. Increased adjusted ROR suggested that dabigatran increases ARs with advanced age; this also supports previous observations on GI hemorrhage reactions related to dabigatran 26.

We provided insights into the association between hemorrhagic reactions and sex in the SRS dataset—the results obtained after adjusting the ROR suggested that sex (female) may influence hemorrhagic reactions. Sex-specific differences in creatinine clearance and renal function exist 22. Creatinine clearance, and presumably renal clearance of drugs, in women tends to be approximately 85% of that in men of the same age and body weight 40. As women generally have less renal function, the risk of hemorrhagic reaction might be considered higher in females than in males. The interaction of the majority of DOACs and sex (female) in GI hemorrhage was observed in both the FAERS and JADER databases. Although the doses of DOACs are optimized according to the renal function of each patient based on the established guideline and package insert, the sex difference observed in our study should be evaluated further.

After a single 20 mg apixaban administration, the mean C_max_ and AUC_∞_ were 18% and 15% higher, respectively, in females than in males 41. As no clinically meaningful age- or sex-related difference in the pharmacokinetics and pharmacodynamics of apixaban has been reported, apixaban is considered safe and well tolerated in both elderly and young subjects of both sexes 41. Previously, dose adjustment was not required on the basis of body weight, age, or sex alone 41,42. However, our results suggest that caution is warranted in the presence of unknown additional factors such as renal impairment that could increase GI hemorrhage risk in females.

For rivaroxaban-associated GI hemorrhage, aging (over 60 years) showed no effects in both the FAERS and JADER databases. It has been previously reported that the influence of age and sex on rivaroxaban therapy was small 43. Neither age nor sex appeared to significantly influence the E_max_, time course of inhibition of Factor Xa activity, or prolongation of prothrombin time 43. Rivaroxaban has been approved for clinical use without dose-adjustment for age or sex alone 43. Clinical studies have demonstrated that the half-life of rivaroxaban is 7-11 h and 11-13 h for young and elderly patients 44-46. In the J-ROCKET-AF study, 15 mg rivaroxaban instead of 20 mg was used 47, because in Japanese patients, 15 mg rivaroxaban provided exposures comparable to 20 mg dose in Americans 48. The trial results demonstrated that 15 mg rivaroxaban was non-inferior as compared with warfarin and it lowered intracranial bleeding, suggesting the use of a reduced dose of rivaroxaban (15 mg) for evaluation in Japanese patients with AF 47. Our study supports the results of these previous reports.

It was difficult to interpret the data of edoxaban, because the number of case reports was small in the FAERS. Edoxaban exposure is affected by the efflux transporter (P-glycoprotein) inhibitors and inducers (amiodarone, quinidine, ketoconazole) 49-51. The effect of concomitantly administered drugs should be investigated in the future.

We also applied time-to-onset analysis to validate the results, which provided novel insights into the time-to-onset of GI hemorrhage, that is, over 30% of GI hemorrhage was observed after 30 days in the real-world data set.

We evaluated nervous system hemorrhage as intracranial hemorrhage. It has been reported that Asians are prone to intracranial hemorrhage 52. For all DOACs, the reporting rates in JADER were higher than those in FAERS. Sex is likely to be a significant factor in DOAC therapy. The interaction terms with DOACs (apixaban, rivaroxaban, and dabigatran) and sex (female) were significant in the FAERS, but not in the JADER.

From the reports of early post-marketing phase vigilance in Japan, severe hemorrhagic ARs in first one month after the use of apixaban, rivaroxaban, and dabigatran were 56.0%, 85.2%, and 56.5%, respectively 53-55. The time-to-onset profile showed that more than 50% of nervous system hemorrhage was observed after 30 days.

Asian patients tended to have lower body weight, lower proportions of prior myocardial infarction, vitamin K antagonist experiences, and concomitant use of gastric antacid drugs, and higher proportion of impaired renal function, prior stroke, nonparoxysmal atrial fibrillation, and antiplatelet medication use 56-58. The differences in safety and efficacy between Asian and Caucasians should be carefully evaluated in the future.

The analysis using SRS such as the FAERS and JADER databases has several notable limitations. The SRS is subject to over-reporting, under-reporting, missing data, exclusion of healthy individuals, lack of a denominator, and the presence of confounding factors 36. However, reports in the SRS databases could also reflect real-life scenarios. The duration of surveillance period could have been increased to strengthen the data obtained in this study. This might be a future consideration. Nomura et al. reported that there were differences in the reported number of ARs between the FAERS and JADER; however, the number of shared reports between the FAERS and JADER is unknown 59. It is improbable to evaluate the “true” risk of ARs without information concerning the total number of patients administered DOACs. In general, ROR cannot be used to infer the comparative strength of causality. However, it offers a rough indication of the signal strength that can be used to generate hypotheses to search for unknown potential ARs 60,61. Patients using any one of the four DOACs (i.e., dabigatran, rivaroxaban, apixaban, and edoxaban) have different risk factors for hemorrhage, and failing to adjust would bias the results. Careful attention must be paid to the interpretation of results. We partially refined the results with a dedicated correction to detect possible confounders present in the database, using multiple logistic regression technique.

## Conclusions

We have reviewed hemorrhagic adverse drug reactions from the SRS databases, real-world registries of patients receiving DOACs. The signals of hemorrhagic reactions such as GI hemorrhage and nervous system hemorrhage were observed in both the SRS databases. Despite the limitations inherent to SRS, we demonstrated that the association of GI hemorrhage induced by DOACs was affected by sex (female). To the best of our knowledge, no time-to-onset analysis of hemorrhagic ARs has been performed using the SRS databases. The aim of the time-to-onset analysis was to obtain new information and compare the risks and onset profiles of hemorrhagic ARs for prescription drugs in the real world. We recommend that female patients who experience symptoms related to GI hemorrhage should be closely monitored and advised to adhere to an appropriate care plan. Additionally, our results show that patients should be closely monitored for hemorrhage even after a month. Results of the present study offer practical considerations for the avoidance and management of GI hemorrhage associated with DOACs. These data will be potentially useful to clinicians for improving the management of ARs associated with DOACs.

## Supplementary Material

Supplementary table.

## Figures and Tables

**Figure 1:**
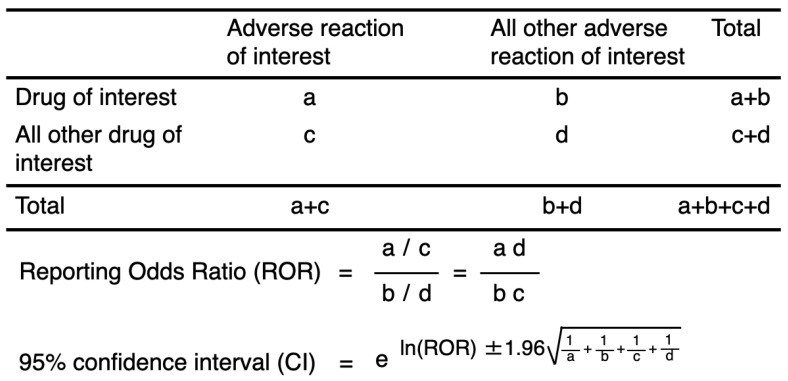
Two-by-two contingency table for calculating the reporting odds ratio.

**Table 1:**
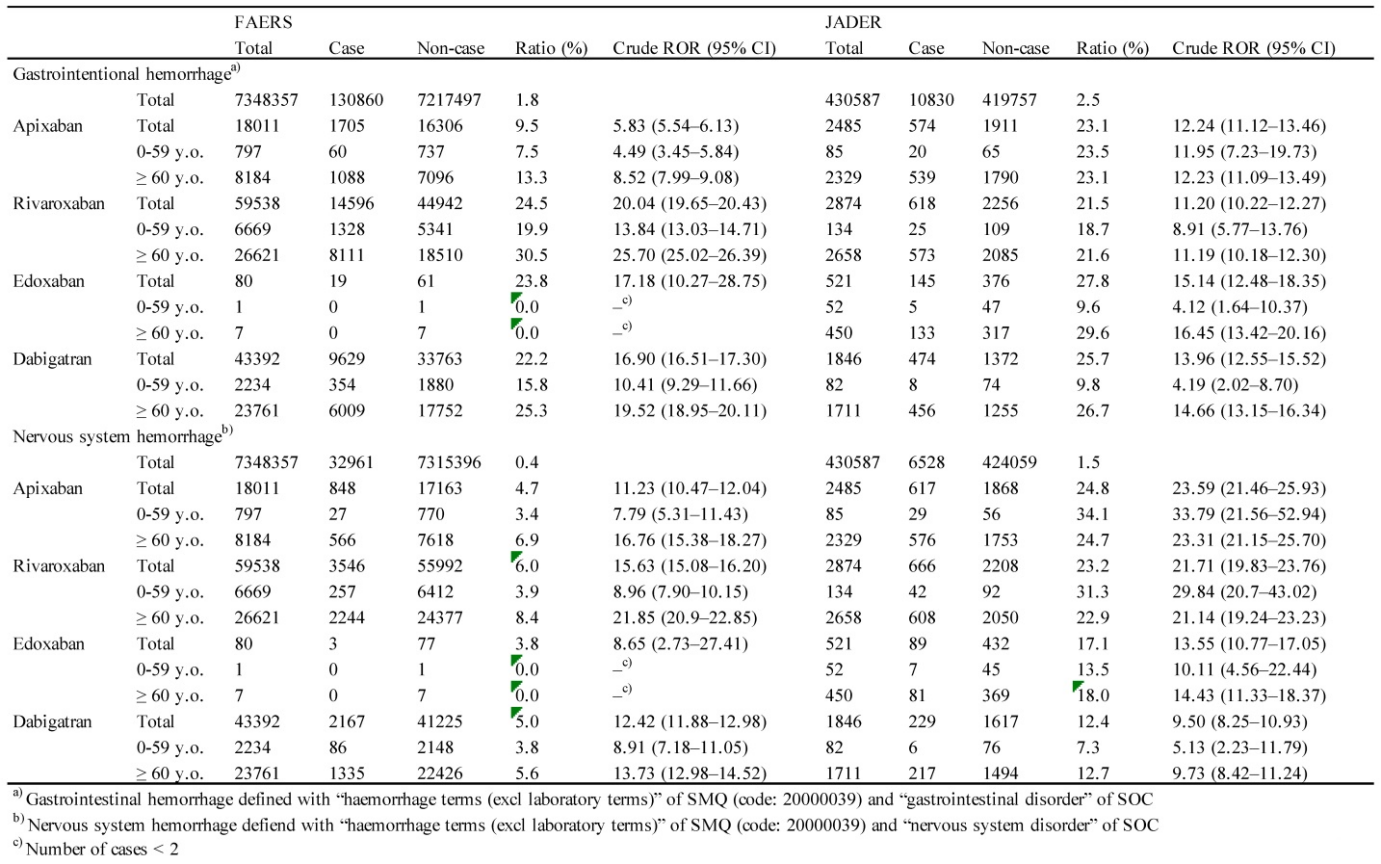
Reported cases and crude ROR of gastrointestinal hemorrhage and nervous system hemorrhage with SMQ code and SOC

**Table 2:**
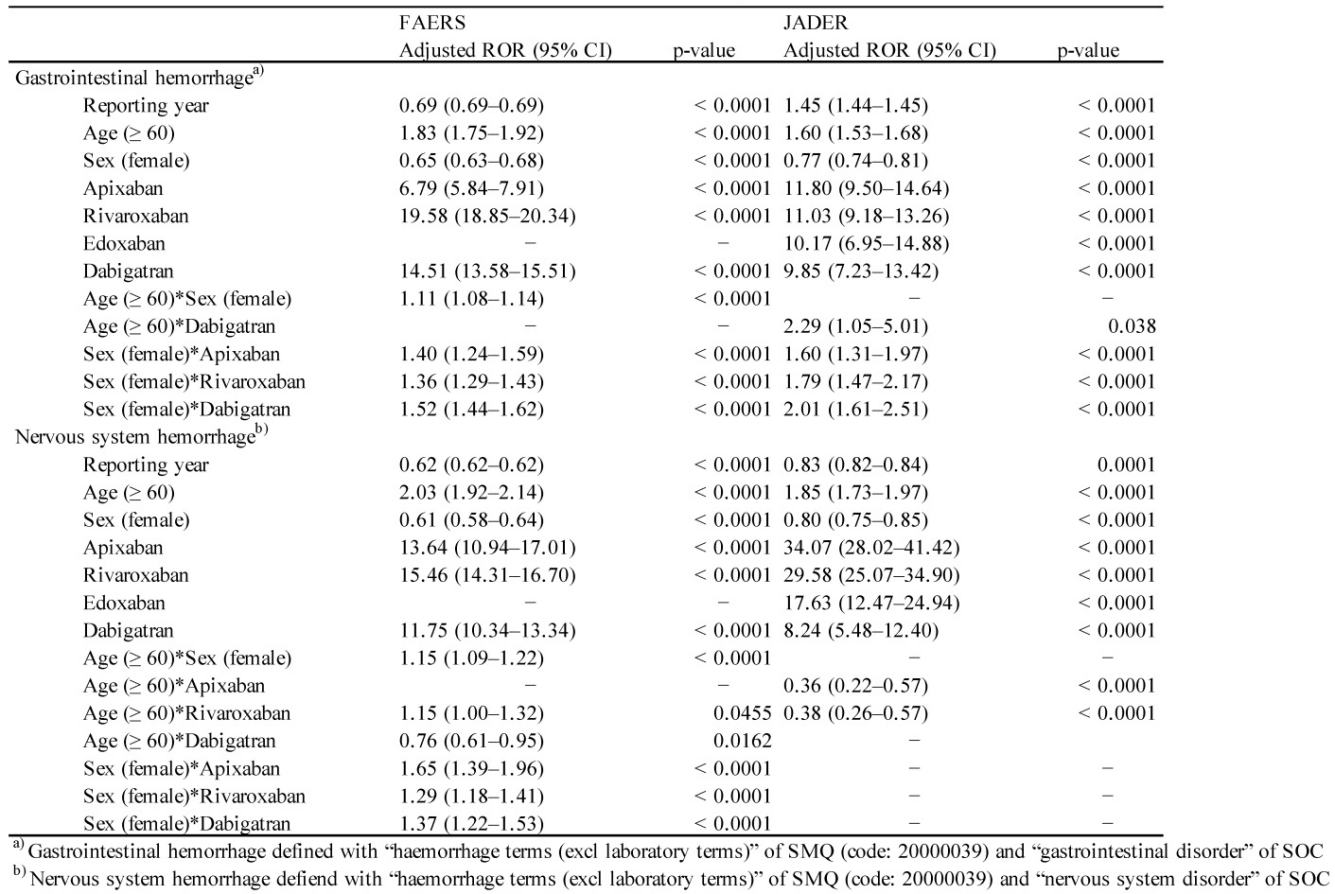
Multiple-logistic regression analysis

## References

[1] Connolly SJ, Ezekowitz MD, Yusuf S (2009). Dabigatran versus warfarin in patients with atrial fibrillation. N Engl J Med.

[2] Patel MR, Mahaffey KW, Garg J (2011). Rivaroxaban versus warfarin in nonvalvular atrial fibrillation. N Engl J Med.

[3] Granger CB, Alexander JH, McMurray JJ (2011). Apixaban versus warfarin in patients with atrial fibrillation. N Engl J Med.

[4] Giugliano RP, Ruff CT, Braunwald E (2013). Edoxaban versus warfarin in patients with atrial fibrillation. N Engl J Med.

[5] Boehringer Ingelheim Pharmaceuticals I. HIGHLIGHTS OF PRESCRIBING INFORMATION. PRADAXA® (dabigatran etexilate mesylate) capsules, for oral use. Accessed 4 Mar 2019.

[6] Janssen Pharmaceutical Companies. HIGHLIGHTS OF PRESCRIBING INFORMATION. XARELTO (rivaroxaban) tablets, for oral use. Accessed 4 Mar 2019.

[7] Bristol-Myers Squibb Company. HIGHLIGHTS OF PRESCRIBING INFORMATION. ELIQUIS (apixaban) tablets for oral use. Accessed 4 Mar 2019.

[8] Daiichi Sankyo Co. L. HIGHLIGHTS OF PRESCRIBING INFORMATION. SAVAYSA (edoxaban) tablets for oral use. Accessed 4 Mar 2019.

[9] Douketis JD, Spyropoulos AC, Spencer FA (2012). Perioperative management of antithrombotic therapy: Antithrombotic therapy and prevention of thrombosis, 9th ed: American College of Chest Physicians Evidence-Based Clinical Practice Guidelines. Chest.

[10] Blech S, Ebner T, Ludwig-Schwellinger E (2008). The metabolism and disposition of the oral direct thrombin inhibitor, dabigatran, in humans. Drug Metab Dispos.

[11] Weinz C, Schwarz T, Kubitza D (2009). Metabolism and excretion of rivaroxaban, an oral, direct factor Xa inhibitor, in rats, dogs, and humans. Drug Metab Dispos.

[12] Raghavan N, Frost CE, Yu Z (2009). Apixaban metabolism and pharmacokinetics after oral administration to humans. Drug Metab Dispos.

[13] Ogata K, Mendell-Harary J, Tachibana M (2010). Clinical safety, tolerability, pharmacokinetics, and pharmacodynamics of the novel factor Xa inhibitor edoxaban in healthy volunteers. J Clin Pharmacol.

[14] Amin A, Deitelzweig S (2015). A case-based approach to implementing guidelines for stroke prevention in patients with atrial fibrillation: balancing the risks and benefits. Thromb J.

[15] Kirchhof P, Benussi S, Kotecha D (2016). 2016 ESC Guidelines for the management of atrial fibrillation developed in collaboration with EACTS. Eur Heart J.

[16] January CT, Wann LS, Alpert JS (2014). 2014 AHA/ACC/HRS guideline for the management of patients with atrial fibrillation: a report of the American College of Cardiology/American Heart Association Task Force on practice guidelines and the Heart Rhythm Society. Circulation.

[17] Miller CS, Grandi SM, Shimony A (2012). Meta-analysis of efficacy and safety of new oral anticoagulants (dabigatran, rivaroxaban, apixaban) versus warfarin in patients with atrial fibrillation. Am J Cardiol.

[18] Capodanno D, Capranzano P, Giacchi G (2013). Novel oral anticoagulants versus warfarin in non-valvular atrial fibrillation: a meta-analysis of 50,578 patients. Int J Cardiol.

[19] Ruff CT, Giugliano RP, Braunwald E (2014). Comparison of the efficacy and safety of new oral anticoagulants with warfarin in patients with atrial fibrillation: a meta-analysis of randomised trials. Lancet.

[20] Desai J, Kolb JM, Weitz JI (2013). Gastrointestinal bleeding with the new oral anticoagulants-defining the issues and the management strategies. Thromb Haemost.

[21] Levey AS, Coresh J, Balk E (2003). National kidney foundation practice guidelines for chronic kidney disease: evaluation, classification, and stratification. Ann Intern Med.

[22] Burton ME, Shaw LM, Schentag JJ (2006). Applied pharmacokinetics & pharmacodynamics: principles of therapeutic drug monitoring. 4th ed.

[23] Bate A, Evans SJ (2009). Quantitative signal detection using spontaneous ADR reporting. Pharmacoepidemiol Drug Saf.

[24] Southworth MR, Reichman ME, Unger EF (2013). Dabigatran and postmarketing reports of bleeding. N Engl J Med.

[25] McConeghy KW, Bress A, Qato DM (2014). Evaluation of dabigatran bleeding adverse reaction reports in the FDA adverse event reporting system during the first year of approval. Pharmacotherapy.

[26] Abe J, Umetsu R, Kato Y (2015). Evaluation of dabigatran- and warfarin-associated hemorrhagic events using the FDA-adverse event reporting system database stratified by age. Int J Med Sci.

[27] Abe J, Umetsu R, Uranishi H (2017). Analysis of polypharmacy effects in older patients using Japanese adverse drug event report database. PLoS One.

[28] Takeyama M, Sai K, Imatoh T (2017). Influence of Japanese regulatory action on denosumab-related hypocalcemia using Japanese adverse drug event report database. Biol Pharm Bull.

[29] Matsui T, Umetsu R, Kato Y (2017). Age-related trends in injection site reaction incidence induced by the tumor necrosis factor-α (TNF-α) inhibitors etanercept and adalimumab: the Food and Drug Administration adverse event reporting system, 2004-2015. Int J Med Sci.

[30] Sauzet O, Carvajal A, Escudero A (2013). Illustration of the weibull shape parameter signal detection tool using electronic healthcare record data. Drug Saf.

[31] Nakao S, Hatahira H, Sasaoka S (2017). Evaluation of drug-induced photosensitivity using the Japanese adverse drug event report (JADER) database. Biol Pharm Bull.

[32] Sasaoka S, Matsui T, Hane Y (2016). Time-to-onset analysis of drug-induced long QT syndrome based on a spontaneous reporting system for adverse drug events. PLoS One.

[33] The Metebolomics Innovation Centre. DRUGBANK. Accessed 4 Mar 2019.

[34] U. S. Food and Drug Administration. “README.DOC” file for the quarterly data extract (QDE) from the FDA adverse event reporting system (FAERS). Accessed 4 Mar 2019.

[35] MedDRA MSSO. Medical Dictionary for Regulatory Activities. Accessed 4 Mar 2019.

[36] van Puijenbroek EP, Bate A, Leufkens HG (2002). A comparison of measures of disproportionality for signal detection in spontaneous reporting systems for adverse drug reactions. Pharmacoepidemiol Drug Saf.

[37] van Puijenbroek EP, Egberts ACG, Heerdink ER (2000). Detecting drug-drug interactions using a database for spontaneous adverse drug reactions: an example with diuretics and non-steroidal anti-inflammatory drugs. Eur J Clin Pharmacol.

[38] Suzuki Y, Suzuki H, Umetsu R (2015). Analysis of the interaction between clopidogrel, aspirin, and proton pump inhibitors using the FDA adverse event reporting system database. Biol Pharm Bull.

[39] Nakamura M, Umetsu R, Abe J (2015). Analysis of the time-to-onset of osteonecrosis of jaw with bisphosphonate treatment using the data from a spontaneous reporting system of adverse drug events. J Pharm Health Care Sci.

[40] Rowland M, Tozer TN, Derendorf H (2011). Clinical pharmacokinetics and pharmacodynamics: concepts and applications. 4th ed.

[41] Frost CE, Song Y, Shenker A (2015). Effects of age and sex on the single-dose pharmacokinetics and pharmacodynamics of apixaban. Clin Pharmacokinet.

[42] Upreti V V, Wang J, Barrett YC (2013). Effect of extremes of body weight on the pharmacokinetics, pharmacodynamics, safety and tolerability of apixaban in healthy subjects. Br J Clin Pharmacol.

[43] Kubitza D, Becka M, Roth A (2013). The influence of age and gender on the pharmacokinetics and pharmacodynamics of rivaroxaban—an oral, direct Factor Xa inhibitor. J Clin Pharmacol.

[44] Kreutz R (2012). Pharmacodynamic and pharmacokinetic basics of rivaroxaban. Fundam Clin Pharmacol.

[45] Kubitza D, Becka M, Wensing G (2005). Safety, pharmacodynamics, and pharmacokinetics of BAY 59-7939—an oral, direct Factor Xa inhibitor—after multiple dosing in healthy male subjects. Eur J Clin Pharmacol.

[46] Kubitza D, Becka M, Roth A (2008). Dose-escalation study of the pharmacokinetics and pharmacodynamics of rivaroxaban in healthy elderly subjects. Curr Med Res Opin.

[47] Hori M, Matsumoto M, Tanahashi N (2012). Rivaroxaban vs. warfarin in Japanese patients with atrial fibrillation - the J-ROCKET AF study -. Circ J.

[48] Tanigawa T, Kaneko M, Hashizume K (2013). Model-based dose selection for phase III rivaroxaban study in Japanese patients with non-valvular atrial fibrillation. Drug Metab Pharmacokinet.

[49] Vranckx P, Valgimigli M, Heidbuchel H (2018). The significance of drug-drug and drug-food interactions of oral anticoagulation. Arrhythmia Electrophysiol Rev.

[50] Mendell J, Zahir H, Matsushima N (2013). Drug-drug interaction studies of cardiovascular drugs involving p-glycoprotein, an efflux transporter, on the pharmacokinetics of edoxaban, an oral factor Xa inhibitor. Am J Cardiovasc Drugs.

[51] Parasrampuria DA, Mendell J, Shi M (2016). Edoxaban drug-drug interactions with ketoconazole, erythromycin, and cyclosporine. Br J Clin Pharmacol.

[52] van Asch CJ, Luitse MJ, Rinkel GJ (2010). Incidence, case fatality, and functional outcome of intracerebral haemorrhage over time, according to age, sex, and ethnic origin: a systematic review and meta-analysis. Lancet Neurol.

[53] Bristol-Myers Squibb company, Pfizer Japan Inc. Early post-marketing phase vigilance of apixaban. Accessed 4 Mar 2019.

[54] Bayer Yakuhin, Ltd. Early post-marketing phase vigilance of rivaroxaban. Accessed 4 Mar 2019.

[55] Boehringer Ingelheim Pharmaceuticals I. Early post-marketing phase vigilance of dabigatran. Accessed 4 Mar 2019.

[56] Hori M, Connolly SJ, Zhu J (2013). Dabigatran versus warfarin: effects on ischemic and hemorrhagic strokes and bleeding in Asians and non-Asians with atrial fibrillation. Stroke.

[57] Wong KSL, Hu DY, Oomman A (2014). Rivaroxaban for stroke prevention in East Asian patients from the ROCKET AF trial. Stroke.

[58] Goto S, Zhu J, Liu L (2014). Efficacy and safety of apixaban compared with warfarin for stroke prevention in patients with atrial fibrillation from East Asia: a subanalysis of the apixaban for reduction in stroke and other thromboembolic events in atrial fibrillation (ARISTOTLE) trial. Am Heart J.

[59] Nomura K, Takahashi K, Hinomura Y (2015). Effect of database profile variation on drug safety assessment: an analysis of spontaneous adverse event reports of Japanese cases. Drug Des Devel Ther.

[60] Montastruc JL, Sommet A, Bagheri H (2011). Benefits and strengths of the disproportionality analysis for identification of adverse drug reactions in a pharmacovigilance database. Br J Clin Pharmacol.

[61] Pariente A, Gregoire F, Fourrier-Reglat A (2007). Impact of safety alerts on measures of disproportionality in spontaneous reporting databases: the notoriety bias. Drug Saf.

