# Supplementary material for: Adverse reaction profiles of hemorrhagic adverse reactions caused by direct oral anticoagulants analyzed using the Food and Drug Administration Adverse Event Reporting System (FAERS) database and the Japanese Adverse Drug Event Report (JADER) database
Source: Int J Med Sci. 2019 Sep 7;16(9):1295–303. doi: 10.7150/ijms.34629 (PMC6775265; doi:10.7150/ijms.34629)
Supplement: Supplementary file 1 — Supplementary table. [file ijmsv16p1295s1.pdf]

Table S1. Reported cases and crude ROR of gastrointentional hemorrhage and nervous system hemorrhage

			FAERS				JADER			
			Total	Case	Non-case	Crude ROR (95% CI)	Total	Case	Non-case	Crude ROR (95% CI)
Gastrointentional hemorrhage[a]										
	Total		7348357	130860	7217497		430587	10830	419757	
	Male		2554275	58678	2495597		211590	6032	205558	
	Female		4092527	60192	4032335		205206	4524	200682	
Apixaban	Total		18011	1705	16306	5.83 (5.54–6.13)	2485	574	1911	12.24 (11.12–13.46)
	Male		7616	761	6855	6.15 (5.71–6.63)	1457	315	1142	10.98 (9.68–12.46)
	Female		7857	740	7117	5.76 (5.34–6.22)	927	238	689	13.67 (11.78–15.86)
	0-59 y.o.	Total	797	60	737	4.49 (3.45–5.84)	85	20	65	11.95 (7.23–19.73)
		Male	419	34	385	4.87 (3.43–6.92)	53	11	42	10.16 (5.23–19.74)
		Female	368	26	342	4.19 (2.81–6.25)	31	8	23	13.49 (6.03–30.17)
	≥ 60 y.o.	Total	8184	1088	7096	8.52 (7.99–9.08)	2329	539	1790	12.23 (11.09–13.49)
		Male	4005	544	3461	8.70 (7.95–9.53)	1355	293	1062	10.96 (9.62–12.49)
		Female	4027	529	3498	8.37 (7.64–9.17)	875	226	649	13.76 (11.82–16.03)
Rivaroxaban	Total		59538	14596	44942	20.04 (19.65–20.43)	2874	618	2256	11.20 (10.22–12.27)
	Male		23386	5925	17461	19.56 (18.98–20.15)	1740	342	1398	9.76 (8.66–11.00)
	Female		24256	5704	18552	17.69 (17.16–18.23)	1051	261	790	13.10 (11.37–15.08)
	0-59 y.o.	Total	6669	1328	5341	13.84 (13.03–14.71)	134	25	109	8.91 (5.77–13.76)
		Male	3050	682	2368	15.96 (14.66–17.39)	96	18	78	8.96 (5.36–14.96)
		Female	3452	595	2857	11.53 (10.56–12.60)	36	7	29	9.36 (4.10–21.37)
	≥ 60 y.o.	Total	26621	8111	18510	25.70 (25.02–26.39)	2658	573	2085	11.19 (10.18–12.30)
		Male	13031	4013	9018	25.29 (24.36–26.26)	1587	307	1280	9.54 (8.41–10.82)
		Female	12970	3873	9097	24.17 (23.27–25.10)	991	251	740	13.43 (11.63–15.52)
Edoxaban	Total		80	19	61	17.18 (10.27–28.75)	521	145	376	15.14 (12.48–18.35)
	Male		2	0	2	0	216	65	151	16.78 (12.54–22.46)
	Female		7	1	6	9.19 (1.11–76.36)	300	79	221	13.95 (10.78–18.05)
	0-59 y.o.	Total	1	0	1	–[c]	52	5	47	4.12 (1.64–10.37)
		Male	1	0	1	–[c]	26	3	23	5.06 (1.52–16.84)
		Female	0	0	0	–[c]	26	2	24	3.23 (0.76–13.67)
	≥ 60 y.o.	Total	7	0	7	–[c]	450	133	317	16.45 (13.42–20.16)
		Male	1	0	1	–[c]	183	61	122	19.48 (14.32–26.51)
		Female	6	0	6	–[c]	262	71	191	14.50 (11.03–19.05)
Dabigatran	Total		43392	9629	33763	16.90 (16.51–17.30)	1846	474	1372	13.96 (12.55–15.52)
	Male		19607	4285	15322	15.91 (15.38–16.47)	1114	245	869	11.16 (9.67–12.87)
	Female		18843	4337	14506	17.02 (16.45–17.62)	685	221	464	18.82 (16.02–22.12)
	0-59 y.o.	Total	2234	354	1880	10.41 (9.29–11.66)	82	8	74	4.19 (2.02–8.70)
		Male	1442	237	1205	10.87 (9.45–12.49)	64	6	58	4.01 (1.73–9.30)
		Female	774	117	657	9.83 (8.07–11.97)	16	2	14	5.54 (1.26–24.37)
	≥ 60 y.o.	Total	23761	6009	17752	19.52 (18.95–20.11)	1711	456	1255	14.66 (13.15–16.34)
		Male	11689	2901	8788	18.60 (17.83–19.40)	1013	236	777	12.01 (10.37–13.91)
		Female	11851	3060	8791	19.63 (18.84–20.47)	659	214	445	18.99 (16.12–22.38)
Nervous system hemorrhage[b]										
	Total		7348357	32961	7315396		430587	6528	424059	
	Male		2554275	15000	2539275		211590	3708	207882	
	Female		4092527	14069	4078458		205206	2623	202583	
Apixaban	Total		18011	848	17163	11.23 (10.47–12.04)	2485	617	1868	23.59 (21.46–25.93)
	Male		7616	382	7234	11.85 (10.68–13.14)	1457	381	1076	24.37 (21.62–27.46)
	Female		7857	368	7489	11.02 (9.92–12.24)	927	210	717	19.63 (16.79–22.93)
	0-59 y.o.	Total	797	27	770	7.79 (5.31–11.43)	85	29	56	33.79 (21.56–52.94)
		Male	419	20	399	11.13 (7.1–17.44)	53	23	30	49.98 (29.01–86.08)
		Female	368	7	361	4.30 (2.04–9.09)	31	6	25	15.60 (6.40–38.05)
	≥ 60 y.o.	Total	8184	566	7618	16.76 (15.38–18.27)	2329	576	1753	23.31 (21.15–25.70)
		Male	4005	260	3745	15.52 (13.68–17.61)	1355	348	1007	23.66 (20.89–26.79)
		Female	4027	290	3737	17.37 (15.40–19.58)	875	202	673	20.09 (17.13–23.55)
Rivaroxaban	Total		59538	3546	55992	15.63 (15.08–16.20)	2874	666	2208	21.71 (19.83–23.76)
	Male		23386	1687	21699	18.13 (17.23–19.08)	1740	430	1310	22.76 (20.35–25.45)
	Female		24256	1429	22827	14.48 (13.71–15.29)	1051	219	832	17.66 (15.18–20.53)
	0-59 y.o.	Total	6669	257	6412	8.96 (7.90–10.15)	134	42	92	29.84 (20.7–43.02)
		Male	3050	151	2899	11.61 (9.85–13.68)	96	33	63	34.19 (22.42–52.14)
		Female	3452	98	3354	6.50 (5.32–7.95)	36	7	29	15.70 (6.87–35.84)
	≥ 60 y.o.	Total	26621	2244	24377	21.85 (20.9–22.85)	2658	608	2050	21.14 (19.24–23.23)
		Male	13031	1166	11865	22.57 (21.23–24.00)	1587	388	1199	22.29 (19.82–25.06)
		Female	12970	1024	11946	19.60 (18.37–20.91)	991	205	786	17.46 (14.94–20.40)
Edoxaban	Total		80	3	77	8.65 (2.73–27.41)	521	89	432	13.55 (10.77–17.05)
	Male		2	0	2	–[c]	216	43	173	16.25 (11.62–22.71)
	Female		7	0	7	–[c]	300	45	255	11.54 (8.39–15.85)
	0-59 y.o.	Total	1	0	1	–[c]	52	7	45	10.11 (4.56–22.44)
		Male	1	0	1	–[c]	26	6	20	19.51 (7.83–48.59)
		Female	0	0	0	–[c]	26	1	25	2.60 (0.35–19.18)
	≥ 60 y.o.	Total	7	0	7	–[c]	450	81	369	14.43 (11.33–18.37)
		Male	1	0	1	–[c]	183	36	147	15.99 (11.1–23.04)
		Female	6	0	6	–[c]	262	44	218	13.19 (9.53–18.26)
Dabigatran	Total		43392	2167	41225	12.42 (11.88–12.98)	1846	229	1617	9.50 (8.25–10.93)
	Male		19607	1000	18607	12.27 (11.50–13.09)	1114	135	979	9.13 (7.61–10.94)
	Female		18843	884	17959	11.20 (10.46–11.99)	685	87	598	9.56 (7.63–11.99)
	0-59 y.o.	Total	2234	86	2148	8.91 (7.18–11.05)	82	6	76	5.13 (2.23–11.79)
		Male	1442	51	1391	8.15 (6.16–10.78)	64	4	60	4.33 (1.57–11.92)
		Female	774	35	739	10.52 (7.49–14.77)	16	1	15	4.33 (0.57–32.79)
	≥ 60 y.o.	Total	23761	1335	22426	13.73 (12.98–14.52)	1711	217	1494	9.73 (8.42–11.24)
		Male	11689	695	10994	14.31 (13.24–15.46)	1013	125	888	9.30 (7.70–11.24)
		Female	11851	618	11233	12.42 (11.45–13.48)	659	86	573	9.87 (7.86–12.39)

[a] Gastrointestinal hemorrhage defined with “haemorrhage terms (excl laboratory terms)” of SMQ (code: 20000039) and “gastrointestinal disorder” of SOC

[b] Nervous system hemorrhage defiend with “haemorrhage terms (excl laboratory terms)” of SMQ (code: 20000039) and “nervous system disorder” of SOC

[c] Number of cases < 2
